# Type III Mixed Cryoglobulinemia and Antiphospholipid Syndrome in a Patient With Partial DiGeorge Syndrome

**DOI:** 10.1080/17402520600877778

**Published:** 2006

**Authors:** Alice D. Chang, Raffi Tachdjian, Kerry Gallagher, Deborah K. McCurdy, Charles Lassman, E. Richard Stiehm, Ora Yadin

**Affiliations:** 1 Division of Allergy, Immunology, and Rheumatology Department of Pediatrics Mattel Children's Hospital University of California Los Angeles CA USA; 2 Department of Pathology and Laboratory Medicine Center for the Health Sciences University of California Los Angeles CA USA; 3 Division of Nephrology Department of Pediatrics Mattel Children's Hospital University of California Los Angeles CA USA

## Abstract

We studied a 14 year-old boy with partial DiGeorge syndrome (DGS), status post complete repair of Tetralogy of Fallot, who developed antiphospholipid syndrome (APS) and type III mixed cryoglobulinemia. He presented with recurrent fever and dyspnea upon exertion secondary to right pulmonary embolus on chest computed tomography (CT). Coagulation studies revealed homozygous methylene tetrahydrofolate reductase 677TT mutations, elevated cardiolipin IgM antibodies, and elevated β_2_-glycoprotein I IgM antibodies. Infectious work-up revealed only positive anti-streptolysin O (ASO) and anti-DNAse B titers. Autoimmune studies showed strongly positive anti-platelet IgM, elevated rheumatoid factor (RF), and positive cryocrit. Renal biopsy for evaluation of proteinuria and hematuria showed diffuse proliferative glomerulonephritis (DPGN) with membranoproliferative features consistent with cryoglobulinemia. Immunofixation showed polyclonal bands. Our patient was treated successfully with antibiotics, prednisone, and mycophenolate mofetil (MMF). This is the first report of a patient with partial DGS presenting with APS and type III mixed cryoglobulinemia possibly due to Streptococcal infection.


*Clinical & Developmental Immunology*, June–December 2006; 13(2–4): 261–264

Taylor & Francis

Taylor & Francis Group

### Type III mixed cryoglobulinemia and antiphospholipid syndrome in a patient with partial DiGeorge syndrome†

ALICE D. CHANG[1], RAFFI TACHDJIAN[1], KERRY GALLAGHER[1], DEBORAH K. MCCURDY[1], CHARLES LASSMAN[2], E. RICHARD STIEHM[1], & ORA YADIN[3]

[1]*Division of Allergy, Immunology, and Rheumatology, Department of Pediatrics, Mattel Children's Hospital, University of California, Los Angeles, CA, USA,* [2]*Department of Pathology and Laboratory Medicine, Center for the Health Sciences, University of California, Los Angeles, CA, USA, and* [3]*Division of Nephrology, Department of Pediatrics, Mattel Children's Hospital, University of California, Los Angeles, CA, USA*

**Abstract**

We studied a 14 year-old boy with partial DiGeorge syndrome (DGS), status post complete repair of Tetralogy of Fallot, who developed antiphospholipid syndrome (APS) and type III mixed cryoglobulinemia. He presented with recurrent fever and dyspnea upon exertion secondary to right pulmonary embolus on chest computed tomography (CT). Coagulation studies revealed homozygous methylene tetrahydrofolate reductase 677TT mutations, elevated cardiolipin IgM antibodies, and elevated $\beta_2$-glycoprotein I IgM antibodies. Infectious work-up revealed only positive anti-streptolysin O (ASO) and anti-DNAse B titers. Autoimmune studies showed strongly positive anti-platelet IgM, elevated rheumatoid factor (RF), and positive cryocrit. Renal biopsy for evaluation of proteinuria and hematuria showed diffuse proliferative glomerulonephritis (DPGN) with membranoproliferative features consistent with cryoglobulinemia. Immunofixation showed polyclonal bands. Our patient was treated successfully with antibiotics, prednisone, and mycophenolate mofetil (MMF). This is the first report of a patient with partial DGS presenting with APS and type III mixed cryoglobulinemia possibly due to Streptococcal infection.

**Keywords:** *DiGeorge syndrome, mixed cryoglobulinemia, glomerulonephritis, antiphospholipid syndrome, homozygous methylene tetrahydrofolate reductase C677T mutations*

### Introduction

DiGeorge syndrome (DGS), first described by Dr Angelo DiGeorge in 1965 (Cooper et al. 1965), now known as the 22q11 deletion syndrome, is associated with craniofacial, cardiac, thymic, immunologic, endocrine, and developmental abnormalities (Perez and Sullivan 2002). Approximately 9–30% of patients with DGS have autoimmune diseases (Jawad et al. 2001; Gennery et al. 2002). Here we describe a patient with DGS who developed cryoglobulinemia and antiphospholipid syndrome (APS).

### Case presentation

A 14 year old Hispanic male with partial DGS presented with recurrent fevers, fatigue, and dyspnea. He had a history of Tetralogy of Fallot, fully repaired at 18 months of age, and a bovine pulmonic valve placement at 8 years of age. Absence of the thymus was noted at the initial surgery. The cardiac history, absent thymus, and 22q11.2 microdeletion led to the diagnosis of DGS. Immunologic evaluation at 18 months of age showed protective titers to *Haemophilus influenzae* and *Diphtheria*, normal lymphocyte subsets, lymphocyte proliferation to Candida but not Tetanus,

Correspondence: A. D. Chang, Mattel Children's Hospital, ULCA, 10833 Le Conte Avenue, MDCC 12-430, Los Angeles, CA 90095, USA. Tel: 1 310 206 1826. Fax: 1 310 825 9832. E-mail: alchang@mednet.ucla.edu

†Grant support: Casey Lee Ball Foundation.

ISSN 1740-2522 print/ISSN 1740-2530 online © 2006 Taylor & Francis
DOI: 10.1080/17402520600877778

and normal quantitative immunoglobulin titers, consistent with partial DGS.

Two months prior to presentation, the patient developed recurrent high fevers, exertional dyspnea, bilateral shin pain, and severe fatigue. Admission temperature was 39°C, with stable vital signs and without respiratory distress on room air. Chest exam revealed clear breath sounds bilaterally, right axillary line tenderness to palpation, and a grade V holosystolic murmur. Routine laboratory evaluation showed a white blood cell count of $5 \times 10^3/\mu l$, hemoglobin of 9.1 g/dl, platelet count of $96 \times 10^3/\mu l$, and sedimentation rate of 32 mm/h (normal < 10). Electrolyte analysis and liver function studies were normal. Urine analysis showed microscopic hematuria (> 180 red blood cells/high power field) with 1 + proteinuria. Chest X-ray showed right lower lobe pneumonia. Chest computed tomography (CT) showed a 1 cm calcified pulmonary embolus in the right branch pulmonary artery with posterior right lower lobe bronchograms. Sinus CT showed pan-sinusitis. Abdominal CT, renal ultrasound, and bone scan were normal. Echocardiogram showed a 65% ejection fraction, mild to moderate pulmonic stenosis, paradoxical septal motion, and absence of vegetations.

Studies for hepatitis A, B and C viruses, Human immunodeficiency virus, Mycoplasma, Cytomegalovirus, Herpes simplex virus, Epstein Barr virus, and *Parvovirus B19* were negative. Anti-streptolysin O (ASO) titers were 385 IU/ml (normal < 150), and anti-DNAse B titers were 1:340 (normal < 1:170). Multiple blood and urine cultures were negative.

Rheumatoid factor (RF) was 143 IU/ml (normal < 25). Anti-platelet IgM was strongly positive. Double stranded DNA (DsDNA) was 261 IU/ml (normal < 200) by enzyme immunosorbent assay, but was negative by indirect immunofluorescence. Antibody titers for Smith, ribonuclear protein (RNP), Sjogren's A and B (SSA, SSB), scleroderma (Scl-70), and anti-neutrophil cytoplasmic antibody (ANCA) were negative. C3 was 85 mg/dl (normal 71–141); C4 was 8 mg/dl (range 12–34). C1Q complexes were 136 μg Eq/ml (normal < 2.0); CH50 was 38 CAE (normal 60–144). The CD3 cells were 802/cmm (normal for age: 1400–2000); CD4 cells were 333/cmm (range 700–1100); CD8 cells were 406/cmm (range 600–900); CD19 cells were 81/cmm (range 300–500). IgG levels were 2190 mg/dl (normal for age: 822–1066), IgA 191 mg/dl (range 85–211), and IgM 228 mg/dl (range 39–59). Serum and urine immunofixation lacked monoclonal bands. Cryocrit was 1% on day 1 (normal: negative on day 4).

Coagulation studies revealed normal thromboplastin and prothrombin times. Dilute Russell viper venom time (DRVVT) was borderline positive; cardiolipin IgM was 17 PLU (normal < 10); $\beta_2$-glycoprotein I ($\beta_2$GPI) IgM was 36 U/ml (normal < 10). Homozygous methylene tetrahydrofolate reductase (MTHFR) 677TT mutations were present with a normal homocysteine level of 5 μmol/l (normal 5–15).

A bone marrow biopsy ruled out malignancy; bacterial, fungal, viral and mycobacterial studies of the marrow were negative. Renal biopsy for evaluation of hematuria and proteinuria (1019 mg/24 h) demonstrated diffuse proliferative glomerulonephritis (DPGN) with features of membranoproliferative glomerulonephritis (MPGN) compatible with cryoglobulinemic glomerulonephritis (Figure 1).

The patient received cefotaxime, azithromycin, heparin followed by warfarin, and prednisone at a dose of 1 mg/kg divided three times daily; within 2 days of prednisone, our patient defervesced for the first time in 3 months. The triad of purpura, weakness, and arthralgia never occurred. The cryocrit resolved within 1 month of prednisone therapy; proteinuria resolved within 2 months. After transient improvement, his fatigue progressed despite treatment with 1 g/day of mycophenolate mofetil (MMF). This resolved completely after successful valvuloplasty for pulmonic stenosis and left pulmonary artery (LPA) stent placement for previously undiagnosed stenosis. After 8 months, patient's ACL IgM was 13 PLU, DRVVT was borderline positive, but $\beta_2$GPI IgM became negative.

In summary, this is a 14 year-old boy with partial DGS who developed APS, type III mixed cryoglobulinemia with DPGN, immune thrombocytopenia, MTHFR 677TT mutations, and pulmonic valve and LPA stenosis.

### Discussion

Up to 30% of patients with DGS have associated autoimmune disease (Jawad et al. 2001; Gennery et al. 2002). These include Graves' disease (Pong et al. 1985; Segni and Zimmerman 2002), juvenile polyarticular rheumatoid arthritis (Sullivan et al. 1997; Davies et al. 2001), Evans syndrome (Pinchas-Hamiel et al. 1994; Kratz et al. 2003), autoimmune hemolytic anemia (Davies et al. 2003), pancytopenia (Davies et al. 2003; Bruno et al. 2002), vitiligo (Jawad et al. 2001), psoriasis (Jawad et al. 2001), vasculitis and Raynaud's (Gennery et al. 2002), and liver disease (Kratz et al. 2003). In these patients, the associated immunologic abnormalities included elevated or low IgG levels, IgA deficiency, decreased T cell function and proliferation, decreased B and T cell subsets. The immunologic anomalies were not consistently associated with any specific autoimmune diagnosis (Pong et al. 1985; Pinchas-Hamiel et al. 1994; Sullivan et al. 1997; Davies et al. 2001; Jawad et al. 2001; Bruno et al. 2002; Gennery et al. 2002; Segni and Zimmerman 2002; Davies et al. 2003; Kratz et al. 2003). Definitive mechanisms for autoimmunity in DGS have not been elucidated to date. The leading theory points to the lack of lymphocyte education and deletion of autoreactive T cells in the thymus

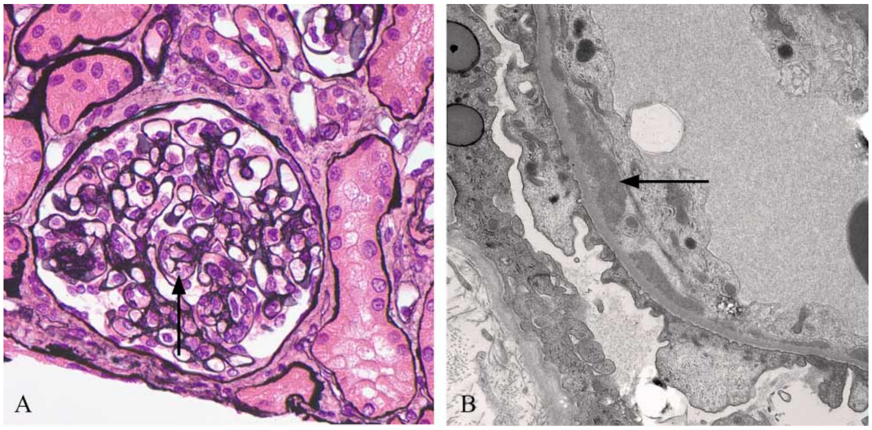

Figure 1. Renal biopsy. A. Jones' methenamine silver stain showed DPGN involving 23 of 25 glomeruli obtained (2 were globally sclerotic), increased cellularity, with prominent glomerular basement membrane (GBM) double contouring (arrow). Mild tubulointerstitial edema and focal tubular atrophy and fibrosis were present. B. Electron microscopy revealed areas of GBM duplication with electron-dense subendothelial deposits (arrow), and endothelial elevation with electron-lucent flocculent material underneath. There is mild mesangial expansion, matrix cellularity, and numerous electron-dense deposits in the paramesangial region. "Fingerprint" substructures within dense deposits and tubuloreticular deposits (seen in systemic lupus erythematosus and HIV nephritis) were absent. Neither vasculitis nor intraluminal thrombi were found. Immunofluorescence (not shown) revealed IgG, IgM, IgA, C1q, C3, κ, and λ staining. This biopsy showing DPGN with features of MPGN was consistent with cryoglobulinemia.

(Gennery et al. 2002). Decreased apoptosis of T cells (Jawad et al. 2001), increased helper to suppressor T cell ratio (Etzioni and Pollack 1994), and oligoclonal T cell expansion (Markert et al. 2004) lead to immune dysregulation and autoimmune phenomena.

Our patient had type III cryoglobulinemia and secondary DPGN. Renal pathology due to mixed cryoglobulinemia varies from focal or mesangial proliferative glomerulonephritis to the predominant type I MPGN (Beddhu et al. 2002). Type III cryoglobulinemia can develop secondary to malignancies, infections (especially Hepatitis B and C), and connective tissue diseases such as APS (Hanly and Smith 2000; Beddhu et al. 2002). Mixed cryoglobulinemia has been associated with Streptococcal infections (Hodson et al. 1978), with RF demonstrating Streptococcal antigen cross-reactivity (Schroder and Christensen 1988). In our patient, elevated ASO and anti-DNase B titers may represent an antecedent group A *Streptococcus* infection or a hypergammaglobulinemic state. It is possible that a Streptococcal infection may have triggered the development of mixed cryoglobulinemia in our patient.

Nucera et al. (2006) reported a patient with DGS who developed APS, thrombocytopenia, cutaneous thrombotic vasculopathy, compound heterozygous MTHFR C677T and A1298C mutations, hyperhomocysteinemia, and cerebral vasculitis. Quantitative immunoglobulin titers and lymphocyte subsets were normal but specific antibody titers and lymphocyte proliferation studies were not reported. Interestingly, our patient also developed APS and had homozygous MTHFR C677T mutations. Thromboembolic risk factors in our patient included stenotic bovine pulmonic valve (Reul et al. 1985), anti-phospholipid antibodies (aPL), and homozygous MTHFR mutations (Haywood et al. 2005). He met both the Sapporo and the newly revised international consensus criteria for APS (Miyakis et al. 2006). Post-infectious aPL production has been described in the literature and may be transient (Shoenfeld et al. 2006). Shoenfeld et al. (2006) showed that mice immunized with *H. influenzae*, *Neisseria gonorrhea*, or Tetanus toxoid produced pathogenic anti-$\beta_2$GPI antibodies that induced thrombocytopenia and fetal loss in healthy pregnant mice. Pathogenic aPL in our patient may have been triggered by a Streptococcal infection. The bovine valve and the MTHFR mutations may have provided the "second hit" in the "two hit" hypothesis, where the "second hit" overcomes the threshold for thrombosis in the presence of aPL ("first hit") (Shoenfeld et al. 2006).

### Conclusion

Autoimmune disease in patients with DGS is relatively common. Our patient and the patient described by Nucera et al. (2006), demonstrate that DGS patients may develop systemic autoimmune disease of significant severity. Given the evidence for the infectious origins of aPL (Shoenfeld et al. 2006), recurrent infections in DGS in the background of compromised immunoregulation may predispose these patients to develop severe autoimmune complications such as APS and cryoglobulinemia. DGS patients are at high risk of infections despite normal immunologic findings (Jawad et al. 2001; Gennery et al. 2002). This is the first report of a patient with partial DGS and MTHFR 677TT mutations who presented with type III mixed cryoglobulinemia and APS after evidence of Streptococcal infection.

### References

Beddhu S, Bastacky S, Johnson JP. 2002. The clinical and morphologic spectrum of renal cryoglobulinemia. Medicine 81:398–409.

Bruno B, Barbier C, Lambilliotte A, Rey C, Turck D. 2002. Autoimmune pancytopenia in a child with DiGeorge syndrome. Eur J Pediatr 161:390–392.

Cooper MD, Peterson RDA, Good RA. 1965. A new concept of the cellular basis of immunity. J Pediatr 67:907–908.

Davies K, Stiehm ER, Woo P, Murray KJ. 2001. Juvenile idiopathic polyarticular arthritis and IgA deficiency in the 22q11 deletion syndrome. J Rheum 28:2326–2333.

Davies JK, Telfer P, Cavenagh JD, Foot N, Neat M. 2003. Autoimmune cytopenias in the 22q11.2 deletion syndrome. Clin Lab Haematol 25:195–197.

Etzioni A, Pollack S. 1994. Autoimmune phenomena in DiGeorge syndrome. Isr J Med Sci 30:853.

Gennery AR, Barge D, O'Sullivan JJ, Flood TJ, Abinun M, Cant AJ. 2002. Antibody deficiency and autoimmunity in 22q11.2 deletion syndrome. Arch Dis Child 86:422–425.

Hanly JG, Smith SA. 2000. Autoimmune antiphospholipid antibodies and cryoglobulinemia. Lupus 9:264–270.

Haywood S, Liesner R, Pindora S, Ganesan V. 2005. Thrombophilia and first arterial ischaemic stroke: A systematic review. Arch Dis Child 90:402–405.

Hodson AK, Doughty RA, Norman ME. 1978. Acute encephalopathy, Streptococcal infection, and cryoglobulinemia. Arch Neurol 35:43–44.

Jawad AF, McDonald-McGinn DM, Zackai E, Sullivan KE. 2001. Immunologic features of chromosome 22q11.2 deletion syndrome (DiGeorge syndrome/velocardiofacial syndrome). J Pediatr 139:715–723.

Kratz CP, Niehues T, Lyding S, Heusch A, Janssen G, Gobel U. 2003. Evans syndrome in a patient with chromosome 22q11.2 deletion syndrome: A case report. Pediatr Hematol Oncol 20: 167–172.

Markert ML, Alexieff MJ, Li J, Sarzotti M, Ozaki DA, Devlin BH, Sempowski GD, Rhein ME, Szabolcs P, Hale LP, Buckley RH, Coyne KE, Rice HE, Mahaffey SM, Skinner MA. 2004. Complete DiGeorge syndrome: Development of rash, lymphadenopathy, and oligoclonal T cells in 5 cases. J Allergy Clin Immunol 113:734–741.

Miyakis S, Lockshin MD, Atsumi T, Branch DW, Brey RL, Cervera R, Derksen RHWM, de Groot DE, Koike T, Meroni PL, Reber G, Shoenfeld Y, Tincani A, Vlachoyiannopoulos PG, Krilis SA. 2006. International consensus statement on an update of the classification criteria for definite antiphospholipid syndrome (APS). J Thromb Haemost 4:295–306.

Nucera C, Vaccaro M, Moleti M, Priolo C, Tortorella G, Angioni A, Ientile R, Violi MA, Loda M, Trimarchi F, Vermiglio F. 2006. Antiphospholipid antibodies syndrome (APS) associated with hyperhomocysteinemia related to MTHFR gene *C677T* and *A1298C* heterozygous mutations in a young man with idiopathic hypoparathyroidism (DiGeorge syndrome). J Clin Endocrinol Metab, Epub.

Perez E, Sullivan KE. 2002. Chromosome 22q11.2 deletion syndrome (DiGeorge and velocardiofacial syndromes). Curr Opin Pedatr 14:678–683.

Pinchas-Hamiel O, Mandel M, Engelberg S, Passwell JH. 1994. Immune hemolytic anemia, thrombocytopenia, and liver disease in a patient with DiGeorge syndrome. Isr J Med Sci 30:530–532.

Pong AJH, Cavallo A, Holman GH, Goldman AS. 1985. DiGeorge syndrome: Long-term survival complicated by Graves disease. J Pediatr 106:619–620.

Reul GJ, Jr, Cooley DA, Duncan JM, Frazier O, Hallman GL, Livesay JJ, Ott DA, Walker WE. 1985. Valve failure with the Ionescu-Shiley bovine pericardial bioprosthesis: Analysis of 2680 patients. J Vasc Surg 2:192–204.

Schroder AK, Christensen P. 1988. Molecular mimicries between human IgG, IgM rheumatoid factor and Streptococcal IgG Fc receptors. Scand J Rheumatol Suppl 75:199–202.

Segni M, Zimmerman D. 2002. Autoimmune hyperthyroidism in two adolescents with DiGeorge/velocardiofacial syndrome (22q11 deletion). Eur J Pediatr 161:233–234.

Shoenfeld Y, Blank M, Cervera R, Font J, Raschi E, Meroni P-L. 2006. Infectious origin of the antiphospholipid syndrome. Ann Rheum Dis 65:2–6.

Sullivan KE, McDonald-McGinn DM, Driscoll DA, Zmijewski CM, Ellabban AS, Reed L, Emmanuel BS, Zackai EH, Arthreya BH, Keenan G. 1997. Juvenile rheumatoid arthritis-like polyarthritis in chromosome 22q11.2 deletion syndromes (DiGeorge anomalad/velocardiofacial syndrome/conotruncal anomaly face syndrome). Arthritis Rheum 40:430–436.

